# TXNIP: A Double-Edged Sword in Disease and Therapeutic Outlook

**DOI:** 10.1155/2022/7805115

**Published:** 2022-04-11

**Authors:** Min Pan, Fengping Zhang, Kai Qu, Chang Liu, Jingyao Zhang

**Affiliations:** ^1^Department of Hepatobiliary Surgery, The First Affiliated Hospital of Xi'an Jiaotong University, Xi'an, Shaanxi 710061, China; ^2^Department of SICU, The First Affiliated Hospital of Xi'an Jiaotong University, Xi'an, Shaanxi 710061, China

## Abstract

Thioredoxin-interacting protein (TXNIP) was originally named vitamin D_3_ upregulated protein-1 (VDUP1) because of its ability to bind to thioredoxin (TRX) and inhibit TRX function and expression. TXNIP is an alpha-arrestin protein that is essential for redox homeostasis in the human body. TXNIP may act as a double-edged sword in the cell. The balance of TXNIP is crucial. A study has shown that TXNIP can travel between diverse intracellular locations and bind to different proteins to play different roles under oxidative stress. The primary function of TXNIP is to induce apoptosis or pyroptosis under oxidative stress. TXNIP also inhibits proliferation and migration in cancer cells, although TXNIP levels decrease, and function diminishes in various cancers. In this review, we summarized the main structure, binding proteins, pathways, and the role of TXNIP in diseases, aiming to explore the double-edged sword role of TXNIP, and expect it to be helpful for future treatment using TXNIP as a therapeutic target.

## 1. Introduction

Thioredoxin-interacting protein (TXNIP) was derived from HL-60 cells stimulated by 1,25-dihydroxyvitamin D3 (1,25-(OH)2D3) [1]. It was initially named vitamin D_3_ upregulated protein-1 (VDUP1) and identified as homologous to VDUP1 using a yeast two-hybrid system [1, 2]. The protein was renamed TXNIP or thioredoxin-binding protein-2 (TBP-2) for its ability to inhibit thioredoxin (TRX) activity and expression [2]. The human TXNIP gene is located on chromosome 1q21.1 with a length of 4174 bp and contains 8 exons [3]. The amino acid sequence of mammalian TXNIP was 96% consistent with that of mice, and a perfect direct repeat sequence was found between the TATA and CCAAT boxes of the mice's TXNIP promoter, suggesting that TXNIP may play a fundamental biological function and be indispensable in mammals [1, 4]. What is more, the human TXNIP is an alpha-arrestin protein with a molecular weight of 46 kDa [3]. TXNIP has a primary structure of 391 amino acids and arrestin-like N-terminal (10–152 aa) and C-terminal (175-298 aa), indicating that its primary function is to inhibit the biological functions of binding proteins [3, 5]. For instance, the C-terminal directly interacts with the E3 ubiquitin ligase ITCH to promote proteasome degradation of TXNIP, as well as the two PPXY motifs at the tail of TXNIP [6].

TXNIP was initially thought to exist only in the cytoplasm. However, several studies found that under oxidative stress, TXNIP could move to diverse intracellular positions. Moreover, the intracellular position of TXNIP, such as mitochondria and the cell surface, may contribute to its biological process [7–10]. In addition, a study has shown that TXNIP is also located in the plasma membrane (PM) [11].

## 2. The Main Binding Protein of TXNIP

### 2.1. Thioredoxin (TRX)

TRX is a 12 kD protein with redox activity. In mammalian cells, TRX has two subtypes: thioredoxin 1 (TRX1) and thioredoxin 2 (TRX2) [12]. TRX2 is found only in mitochondria, while TRX1 is mainly present in the cytosol, PM, nucleus, as well as extracellular space [13]. There are two different antioxidant systems, the cytosolic TRX system and the mitochondrial TRX system. The cytosolic TRX system consists of TRX1, TRX receptor (TRXR), and peroxiredoxin (PRX). However, the mitochondrial TRX system consists of TRX2, TRXR2, and peroxiredoxin-3 (PRX3) [14, 15]. TXNIP interacts with both cytosolic TRX1 and mitochondrial TRX2, indicating that the TRX/TXNIP redox system works in both the cytosol and mitochondria [5]. TXNIP directly binds to reduced TRX1, thereby inhibiting TRX1 reducibility, and acts in a redox-dependent manner, including oxidation, anti-inflammatory, and anticancer effects [13]. In addition, under oxidative stress, TXNIP travels into the mitochondria and binds to TRX2 in the mitochondria, releasing TRX2-bound apoptotic signal-regulated kinase 1 (ASK1), which activates the ASK1-mediated apoptosis pathway [8, 16].

In 2006, Patwari et al. [5] confirmed that the binding of TRX to TXNIP depends on the stable formation between the 32nd cysteine residue (Cys32) of TRX and the 247th cysteine residue (Cys247) of TXNIP. Moreover, it is critical to combine TXNIP and TRX through the TXNIP-Cys63-Cys247-TXNIP intermolecular disulfide bond formed between the two TXNIPs. Based on this research, Hwang et al. [17] reported the crystal structure of TRX and TXNIP interaction in 2014. Through experiments such as coimmunoprecipitation (coimmunoprecipitation, CO-IP) and magnetic resonance (nuclear magnetic resonance, NMR), they deeply revealed the interaction between TRX and TXNIP. The disulfide bond between the two TXNIP molecules broke, allowing TXNIP and TRX to form an intermolecular disulfide bond and, in the same breath, an intramolecular disulfide bond between Cys63 and Cys190 in TXNIP. The disulfide bond conversion mechanism is affected by the oxidation-reduction state [17].

### 2.2. Poly-ADP-Ribose Polymerase 1 (PARP1)

Poly-ADP-ribose polymerase 1 (PARP1) is a nuclear protein, that is affected by the cellular redox state, especially in DNA single-strand breakage induced by oxidative stress [18, 19]. PARP1 binds to the target protein mainly in two ways: the first is to serve as a scaffold protein to bind to the target protein directly or indirectly and make it located in the nucleus; the second is to transfer the ADP-ribose part to the target protein to mediate the localization and function of the target protein [11]. Under transient stimulation of physiological concentrations of hydrogen peroxide (H2O2) or tumor necrosis factor (TNF), TXNIP transfers from the nucleus to the PM, binds to, and activates the vascular endothelial growth factor receptor 2 (VEGFR2) signal to induce apoptosis [11].

## 3. The Core Pathway of TXNIP

**Figure 1:**
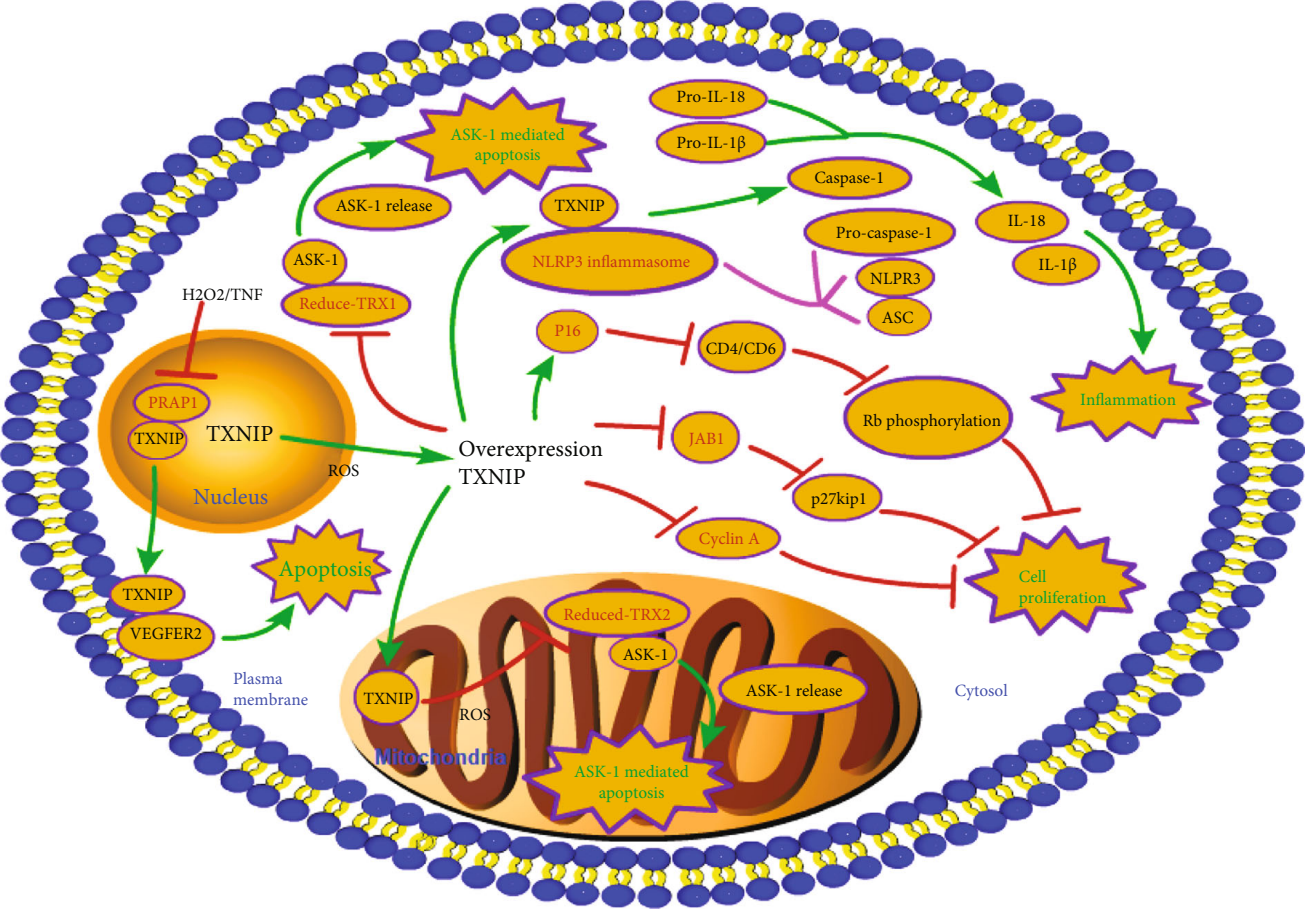
The core pathway of TXNIP. TXNIP: thioredoxin-interacting protein; PRAP1: poly-ADP-ribose polymerase 1; H2O2: hydrogen peroxide; ROS: reactive oxygen species; TNF: tumor necrosis factor; ASK-1: apoptotic signal-regulated kinase 1; TRX1: thioredoxin 1; TXR2: thioredoxin 2; NLRP3: NOD-like receptor family protein 3; pro-caspase-1: pro-cysteinyl aspartate specific proteinase-1; ASC: apoptosis-associated speck-like protein.

The core pathway of TXNIP is shown in Figure 1.

### 3.1. TXNIP and Apoptosis

The primary functions of TXNIP are to induce apoptosis or pyroptosis under oxidative stress. TXNIP acts like a supervisor that kills cells if they cannot tolerate oxidative stress. The interaction between TRX and ASK1 is dependent on the redox state. ASK-1 only binds to reductive TRX. Under normal conditions, TRX inhibits apoptosis signal-regulating kinase 1 (ASK1) kinase activity and ASK1-dependent apoptosis by directly binding to the N-terminus of ASK1 [20]. Under oxidative stress, the TXNIP will be released from the nucleus and bind to TRX1 in the cytosol and TRX2 in the mitochondria [9]. In the cytoplasm, overexpressed TXNIP binds to TRX1 and releases ASK-1, thereby activating the p38 mitogen-activated protein kinase (p38 MAPK) signaling pathway [21–23]. Mitochondria is an important organelle for reactive oxygen species (ROS) production. During oxidative stress, TXNIP is transferred from the nucleus to the mitochondria, where it binds to TRX2 and releases ASK-1, leading to apoptosis [8, 23]. In addition, TXNIP can also bind to NLRP3 inflammasomes and induce pyroptosis by stimulating the secretion of interleukin-1*β* (IL-1*β*) dependent on the NLRP3 ASC-Caspase-1 pathway [24].

### 3.2. TXNIP and Inflammation and Immune Reaction

The NLRP3 inflammasome is an intracellular polyprotein complex, as one of the components encoding the pattern recognition receptor (PRR) inflammasome. It mainly includes NOD-like receptor family protein 3 (NLRP3), apoptosis-associated speck-like protein (ASC), and pro-cysteinyl aspartate specific proteinase-1 (pro-caspase-1), which play a crucial role in inflammation and immune response by activating inflammatory caspase-1 [25]. The formation of the NLRP3 inflammasome can be divided into three stages: the initiation stage, activation stage, and oligomerization stage [26, 27]. The initial stage is mainly the transcription of NLRP3 inflammatory body components. In most cases [27], the initiation is NF downstream of pattern recognition receptor- *κ*B (NF-*κ*B) activation, which provides other ligands such as TOLL-like receptor (TLR) and NLR (such as NOD1 and NOD2) [28]. In the activation and oligomerization stages, NLRP3 is stimulated by danger signals, such as ROS [24], K^+^ outflow [29], and crystal deposition [25], and binds to ASC to recruit pro-caspase-1. The recruited pro-caspase-1 forms an oligomer, which can be cut into P20 and P10 subunits after self-catalysis. Caspase-1 is composed of two groups of such subunits; mature caspase-1 mediates IL-1*β* and IL-18, which is the critical process of the immune response. TXNIP was identified as the potential binding partner of NLRP3 in a yeast two-hybrid system with leucine-rich repeats (LRRs) as bait [30, 31]. Studies have shown that under oxidative stress, TXNIP is released from the oxidized TRX and binds to the NLRP3 inflammasome, which activates and releases IL-1*β* and IL-18, thereby, inducing an inflammatory immune response [24].

### 3.3. TXNIP and Proliferation and Aging

Studies have shown that TXNIP gene expression is closely related to the cell cycle process. Overexpression of TXNIP inhibited the activity of the cyclin A promoter and induced cell cycle arrest in *G*_0_/*G*_1_ [32]. In addition, some cell cycle factors also relate to TXNIP-mediated cell cycle arrest. P16 primarily binds to and inhibits cyclin-dependent kinases 4 (CDK4) and cyclin-dependent kinases 6 (CDK6) and inhibits G_1_-_S_ transition by decreasing phosphorylation of Rb, which controls growth inhibition properties. When TXNIP is overexpressed in HTLV-I positive T cells, p16 expression is increased, which reduces Rb phosphorylation and prevents cells from entering the S phase [33]. p27^kip1^, another cell cycle regulator, is also associated with TXNIP-mediated cell cycle arrest confirmed in the deficient mouse model [34–36]. The stability of p27^kip1^ is regulated by the JAB1 gene [37]. TXNIP overexpression interacts with JAB1 and inhibits JAB1-mediated p27^kip1^ degradation and cell proliferation [34].

## 4. The Role of TXNIP in Disease

TXNIP-mediated oxidative stress plays an essential regulatory role in the initiation and development of diabetes, and others. As a result, many TXNIP-related target drugs have emerged.

### 4.1. Diabetes and Its Complications

**Figure 2:**
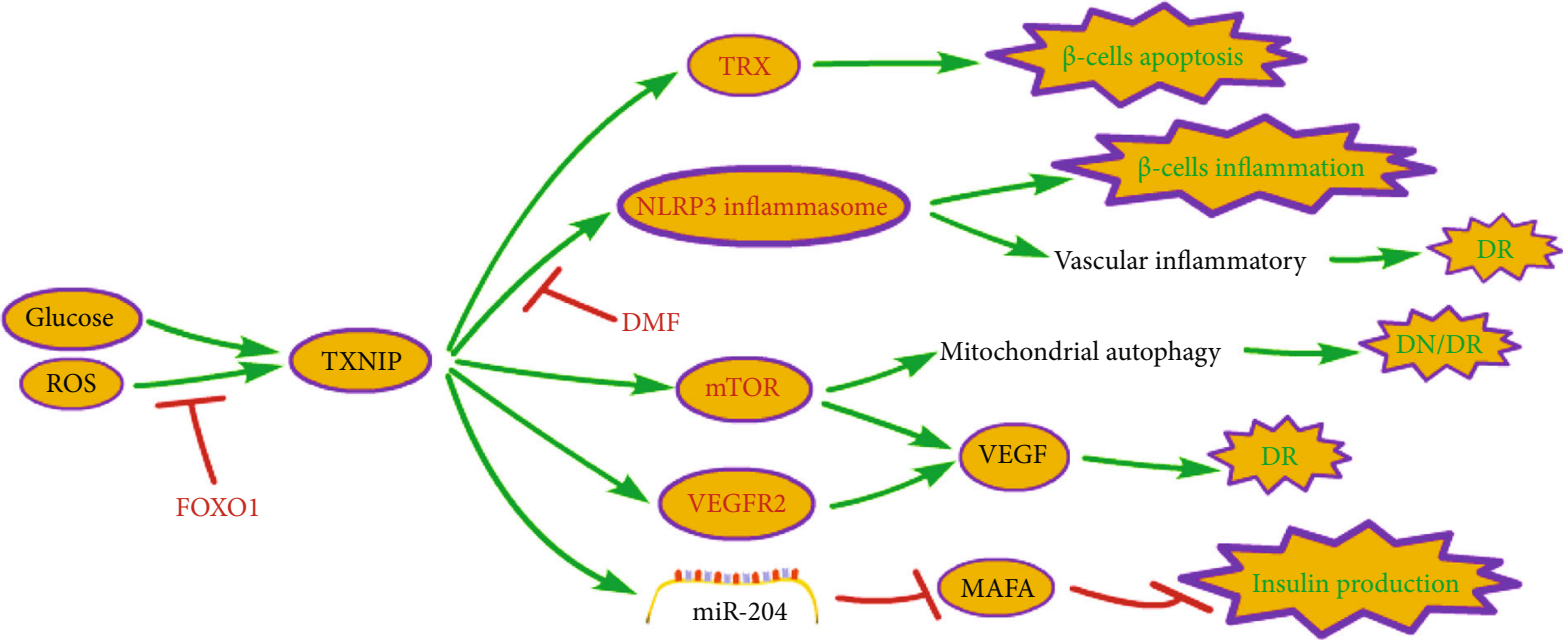
The main mechanism of TXNIP in diabetes. ROS: reactive oxygen species; TXNIP: thioredoxin-interacting protein; TRX: thioredoxin; NLRP3: NOD-like receptor family protein 3; DMF: dimethyl fumarate; VEGFER2: vascular endothelial growth factor receptor 2; VEGF: vascular endothelial growth factor; DR: diabetic retinopathy; DN: diabetic nephropathy.

Diabetes is a chronic metabolic disease caused by various etiologies, mainly manifested by chronic hyperglycemia. Pancreatic *β*-cells are of utmost importance in maintaining glucose homeostasis by sensing blood glucose levels and secreting insulin to regulate metabolism. Sustained high blood glucose levels can have deleterious effects on many organ systems. In the pancreas, high-glucose toxicity leads to impaired insulin gene transcription [38] and irreversibility due to apoptosis *β* cell loss [39–45], which leads to the vicious circle of hyperglycemia deterioration. The main mechanism of TXNIP in diabetes is shown in Figure 2.

TXNIP levels increased significantly in diabetic patients and those with chronic hyperglycemia, suggesting that TXNIP levels are bound up with the development and progression of diabetes [46]. Isolated intact human islets cultured under low-glucose and high-glucose conditions were analyzed by oligonucleotide microarray, the expression of the TXNIP coding gene increased 11 times more than the glucose-induced expression group [47]. Subsequently, the upregulation of TXNIP in INS-1 cells and primary human islets was verified, and it was found that the glucose-induced TXNIP response was mediated by cis-acting elements, including conserved E-box repeats in the TXNIP promoter and transacting factor carbohydrate response element-binding protein (ChREBP) [48]. Furthermore, it was discovered that an increase in TXNIP level over time is not only a result of an increase in blood glucose level and/or endoplasmic reticulum (ER) stress, but it is also a part of the vicious circle [49]. It can stimulate its expression through the positive feedback loop involving the activation of ChREBP and amplify the effect of adverse effects related to cell biology, including oxidative stress and inflammation, eventually leading to *β* cell death and disease progression [49].

Increased TXNIP expression is pertinent to *β*-cell apoptosis. TXNIP-overexpressing INS-1 cells were found to be significantly more susceptible to apoptosis than lacZ-overexpressing control cell lines [48]. The level of TXNIP protein increased by nearly 10 times that of the normal glucose control group in obese mice. TXNIP induces IL-1*β* production by activating the NLRP3 inflammasome and IL-1*β* mRNA transcription, leading to *β*-cell death [50]. In addition, increased TXNIP expression can directly control insulin production in the body. It was found that miR-204 expression in INS-TXNIP cells was higher than that in INS-LacZ cells [51]. Further study founded that TXNIP induced *β* cells express miR-204 and reduced insulin production by directly targeting and downregulating the insulin transcription factor MAFA (the newly identified TXNIP-miR-204-MAFA-insulin pathway) [51].

As a specific complication of diabetes, microangiopathy is characterized by microcirculatory disturbance and microvascular basement membrane thickening. It involves all tissues and organs of the body, mainly the retina, kidneys, nerves, and myocardium. When ROS is elevated, it can induce long-term vascular complications by upregulating serum and tissue TXNIP levels, activating NF-*κ*B and NLRP3 inflammasome pathways, and increasing the expression of inflammatory cytokines. Dimethyl fumarate (DMF) may destroy ROS-TXNIP and/or ROS-NF-*κ*B pathways and inhibit the activation of NLRP3 inflammatory corpuscles in the diabetic aorta and prevent vascular complications in diabetic patients [52]. Diabetic retinopathy (DR) is the leading cause of blindness in the working class worldwide. TXNIP deficiency inhibits angiogenic responses induced by VEGF or/and moderately high glucose (MHG) in human retinal microvascular endothelial cells (HRMECs) and mouse retinas, by inhibiting the activation of VEGFR2 and Akt/mTOR pathways in HRMECs, MHG induced ROS generation [53]. It is reported that mitochondrial autophagy disorder and activation of the NLRP3 inflammasome will accelerate DR [54, 55]. Diabetic nephropathy (DN) is one of the most severe complications of diabetes and the leading cause of end-stage renal disease. A study found that TXNIP leads to dysregulation of renal tubule and mitochondrial autophagy in DN through activation of the mTOR signaling pathway, contributing to the progression of DN [56]. But the activation and regulation of FOXO1/TXNIP-TRX in DN can protect renal proximal tubular cells from high glucose-induced injury by reducing the production of ROS [57].

### 4.2. Cardiovascular Disease

**Figure 3:**
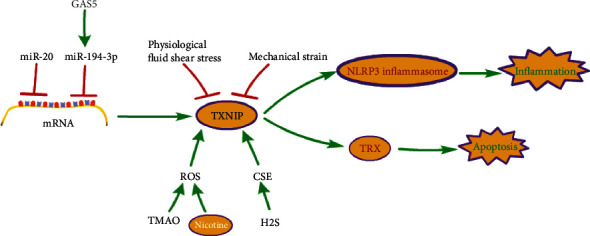
The main mechanism of TXNIP in cardiovascular disease.

Cardiovascular disease is a chronic disease closely associated with aging [58]. Atherosclerosis (AS) is the main cause of cardiovascular disease. Endothelial cell (EC) aging related to atherosclerosis is accompanied by the destruction of endothelial cell integrity and the functional damage of endothelial cells, leading to vascular dysfunction and creating conditions for the occurrence of cardiovascular diseases [59, 60]. Oxidative stress [61–63], ER stress [61–63], and inflammation [64, 65] have become the main harmful factors leading to endothelial cell dysfunction. When oxidative stress occurs, the vascular wall will produce excessive ROS, which will damage the structure and function of ECs and enhance the inflammatory response of the vascular wall. The activation of the inflammasome in macrophages [66] and ER [67] has been considered an essential step in the progression of AS. The main mechanism of TXNIP in cardiovascular disease is shown in Figure 3.

Under oxidative stress, the increase of TXNIP expression leads to the cell aging of endothelial cells. Foam cells are the primary sources of ROS. By using the coculture Transwell system to detect lipid peroxidation products secreted by foam cells produced by macrophages after OxLDL exposure, such as 4-hydroxynonenal (4-HNE) activating nuclear factor PPAR-*δ*, translocation of the TXNIP promoter leads to increased TXNIP expression, which promotes EC aging [68]. In recent years, micro-RNSs have been regarded as therapeutic targets and biomarkers for evaluating cardiovascular diseases [69]. The low expression of miR-20b can downregulate the level of cell aging markers, thus showing the role of miR-20 in maintaining vascular integrity [70]. The high expression of miR-20b can inhibit endothelial cell aging, which may be through TXNIP/NLRP3 axis to inhibit Wnt/*β*-catenin path implementation [71]. In addition, a study has confirmed that depleted GAS5 inhibits TXNIP by upregulating miR-194-3p, promotes the growth of vascular ECs, and reduces the formation of atherosclerotic plaque in AS [72].

The production of ROS activates TXNIP-NLRP3, which is involved in the pathogenesis of the cardiovascular disease [73]. There is increasing evidence that AS is mediated by inflammasomes [74]. The risk factors are the plasma levels of the choline-derived metabolite trimethylamine-N-oxide (TMAO) [75], the plasma level of hydrogen sulfide (H2S) in hemodialysis patients [76], and smoking [77, 78]. TMAO and nicotine have been identified as accelerators [79, 80], with TMAO inducing oxidative stress in human umbilical vein endothelial cells (HUVEC), activating the ROS-TXIP-NLRP3 inflammasome signal transduction, and releasing inflammatory factors, leading to endothelial dysfunction [67]. The proatherosclerotic property of nicotine is to increase the production of ROS, activating the TXNIP/NLRP3 inflammasome signal and causing macrophage pyrolysis [81]. H2S produced by cystathionine *γ*-lyase (CSE) and passed through the H2S/CSE-TXNIP-NLRP3-IL-18/IL-1*β*-NO signaling pathway, on the contrary, inhibits TXNIP function and plays a role in AS [82].

AS is characterized by a blood flow disorder [83–85]. A large number of studies show that physiological fluid shear stress plays a protective role in atherosclerosis because atherosclerosis preferentially occurs in areas with flow disorder or low shear stress, while areas with stable laminar flow and physiological shear stress are protected [86, 87]. Mechanical strain inhibits the expression of TXNIP and increases TRX activity, which has been confirmed in cardiomyocytes [88]. Furthermore, TXNIP expression is reduced during TRX-induced cardiomyocyte growth stimulation following pressure overload myocardial hypertrophy [89]. The protective effect of physiological fluid shear stress on AS is to decrease TXNIP expression and increase TRX expression, thereby limiting the proinflammatory events mediated by the TNF-ASK1-JNK/p38 pathway [90].

### 4.3. Cancer

**Figure 4:**
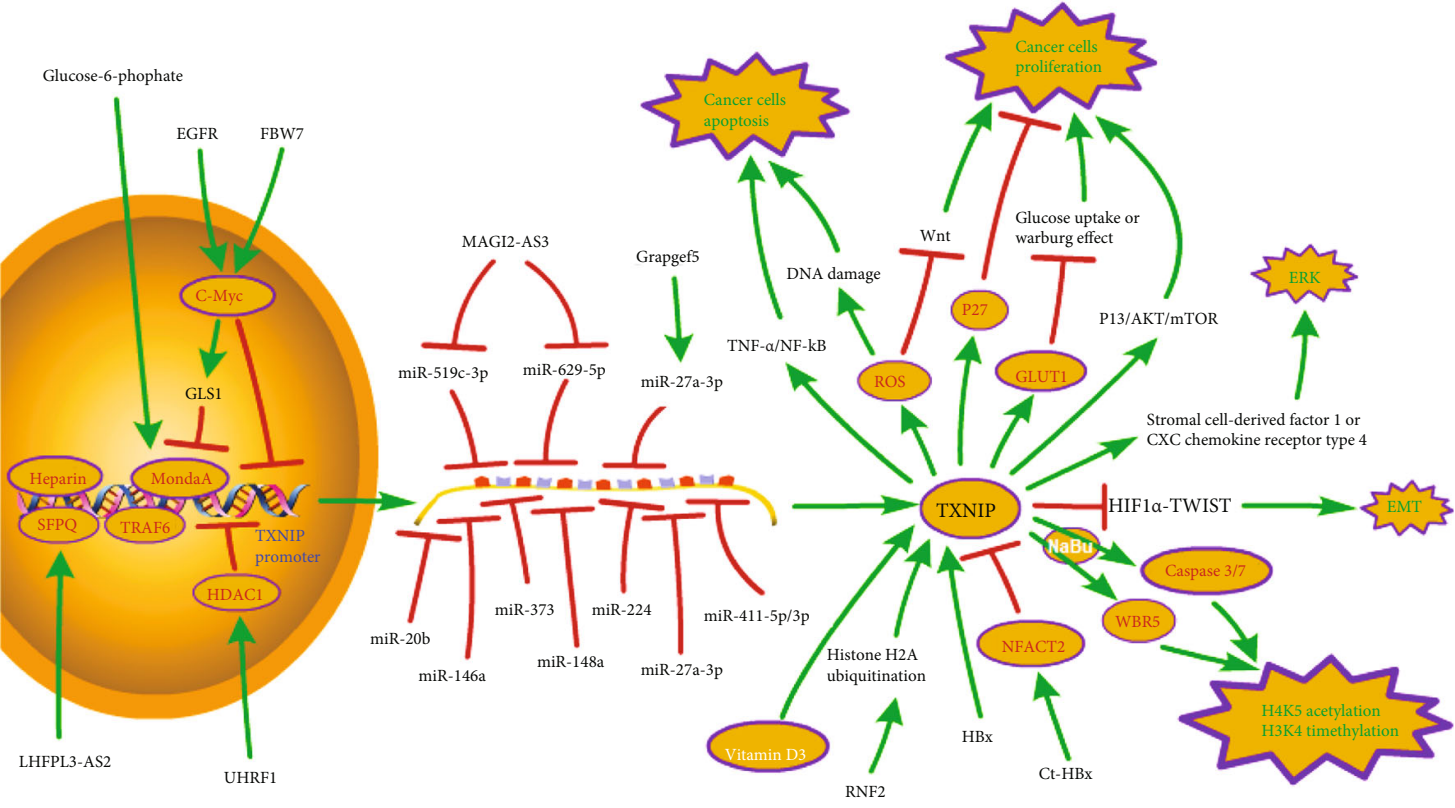
The main mechanism of TXNIP in cancer. TXNIP: thioredoxin-interacting protein; TRAF6: TNF receptor-related factor 6; GLS1: glutaminase 1; HDAC1: histone deacetylase 1; UHRF1: ubiquitin-like PHD and ring finger domain 1; FBWT7: F-box and WD repeat domain 7; Grapgef5: circRNA rapef5; HBx: hepatitis B virus X; Ct-HBx: C-terminal truncated X protein; NF-*κ*B: NF downstream of pattern recognition receptor-*κ*B; NFACT2: nuclear factor of activated T cells 2; HIF1*α*: hypoxia-inducible factor 1*α*; NaBu: sodium butyrate; EMT: epithelial-mesenchymal transition; ERK: extracellular signal-regulated kinase.

TXNIP is considered to be a potential tumor suppressor gene. Many studies have shown that the expression of TXNIP is at a low level in different types of cancer (such as liver cancer, breast cancer, and lung cancer), and the overexpression of TXNIP inhibits the proliferation of cancer cells. The main mechanism of TXNIP in cancer is shown in Figure 4.

The prognosis and predictive ability of TXNIP in human breast cancer have been confirmed. The expression of TXNIP in breast cancer tissues significantly decreases as the tumor progresses. The miR-373-TXNIP-HIF1*α*-TWIST signaling axis as well as EGFR_high_-MYC_high_ -TXNIP_low_ signature shows more aggressive cancer features and can be used as independent prognostic factors for patients with breast cancer [91, 92]. TXNIP influences the outcome of breast cancers through several mechanisms: (1) TXNIP induces the expression of p27 and inhibits the TXNIP-ROS-Wnt pathway to inhibit the proliferation of breast cancer cells [93, 94]. (2) TXNIP impedes the epithelial-mesenchymal transition (EMT) and metastasis by downregulating HIF1-*α* and subsequently decreasing the expression of TWIST which is a core factor for tumor EMT and metastasis [91, 95]. (3) TXNIP hampers the glucose uptake by GLUT-1 inhibition and the Warburg effect, which reduces the ATP synthesis against high energy demand for protein synthesis for cell proliferation [93, 96]. (4) TXNIP binds to TRX protein accordingly increasing the level of ROS within cancer cells which causes ROS-mediated DNA damage, inducing cell apoptosis. This effect preferentially occurs in BRCA1-/- breast cancer cells with a compromised DNA repair system [97]. (5) Some researchers found that low TXNIP expression is associated with a worse prognosis in node-negative breast cancer, and the expression level of TXNIP is influenced by the ERBB2. [98]. TXNIP levels within cancer cells can be regulated by many factors. The expression of TXNIP is strongly, if not entirely, dependent on the MondoA transcription factor [99]. MondoA binds a double E-box carbohydrate response element (ChoRE) in the TXNIP promotor and promotes the expression of TXNIP, and it is a sensor of a high cellular energy charge. The high intracellular glucose-6-phosphate drives translocation of MondoA from the outer mitochondrial membrane (OMM) to the nucleus where MondoA binds to the promotor of TXNIP and recruits cofactors that initiate transcription [100–102]. Hyperglycemia itself has the ability to affect the level of TXNIP RNA. Besides the glucose, the EGFR-My-TXNIP axis can also regulate the expression of TXNIP. C-Myc binds to an E-box-containing region in the TXNIP promotor, competing with MondoA to inhibit the TXNIP expression [103]. Furthermore, micro-RNAs like miR-146a, miR-148a, as well as miR-373 bind to the 3′UTR of TXNIP mRNA and participate in the negative regulation of TXNIP expression [104–106].

A study [107] showed that TXNIP expression in human hepatocellular carcinoma (HCC) specimens and HCC-derived cell lines is low or absent. Chronic hepatitis B virus (HBV) infection is a primary cause of the progression of HCC. The hepatitis B virus X (HBx) protein is a multifunctional protein encoded by the HBx gene. Some studies claimed that the overexpression of TXNIP strengthens the migration and invasion of HepG2 cells in the transfer of HCC related to hepatitis B, suggesting that HBx-mediated HBV-associated HCC can promote TXNIP expression [108]. Others showed that the C-terminal truncated X protein (Ct-HBx) produced by the preferential clustering pattern at the 3′-end of the HBV genome X gene downregulates TXNIP by activating transcriptional repressor nuclear factor of activated T cells 2 (NFACT2), reprogramming glucose metabolism to initiate HCC [109]. In addition, the miR-519c-3p [110] and M2 macrophage-derived exosomal miR-27a-3p [111] promote the HCC progression by downregulating TXNIP, while MAGI2-AS3 inhibits the HCC progression through miR-519c-3p/TXNIP axis [110]. What is more, in patients with chronic liver disease, vitamin D_3_ can stimulate the expression of TXNIP, which may reduce the carcinogenic effect [107]. It has been shown that heparin can affect cell growth, differentiation, invasion, and migration by binding to the ChoRE-b site one of the TXNIP promoters of TXNIP and promoting the transcription of TXNIP in HCC cells [112, 113]. Pancreatic ductal adenocarcinoma (PDAC) is the dominating cause of pancreatic cancer-specific death [114]. MicroRNAs (miRNAs) are involved in developing PDAC by regulating numerous cellular processes. MiR-224 reverses modulate TXNIP by directly binding to the TXNIP 3′-untranslated region, activating the hypoxia-inducible factor 1*α* (HIF1*α*) [115]. Also, TXNIP reexpression or HIF1*α* depletion eliminated the effects of miR-224 on PDAC cell internal and external proliferation and migration [115]. In addition, another study has shown that F-box and WD repeat domain 7 (FBW7) act as negative regulators of glucose metabolism by regulating the C-Myc/TXNIP axis in pancreatic cancer, thereby inhibiting the occurrence and development of tumors [116]. Knocking out the TXNIP gene in a mouse model is related to helicobacter pylori-associated gastric cancer [117]. The expression level of TXNIP was meaningfully lower in gastric cancer (GC) tissues compared with normal tissues, TXNIP negatively regulates helicobacter pylor-related GC by inhibiting TNF*α*-induced activation of NF-*κ*B signaling pathway [117]. miR-20b inhibits TXNIP, which is involved in the PI3K/AKT/mTOR pathway to promote the GC progression by mediating glucose uptake in cancer cells [118]. TXNIP may be a therapeutic target of Weining granule (WNG) for GC [119].

The study has found that inhibition of PI3K/AKT signaling by tyrosine kinase inhibitors (TKI) leads to increased TXNIP expression in non-small-cell lung cancer (NSCLC) tissue [120]. MiR-411-5p/3p [121] and miR-629-5p [122] were upregulated in human NSCLC tissues, and the overexpression of both could inhibit the expression of TXNIP, promoting tumor migration and proliferation and preventing cell apoptosis in NSCLC cell lines, while a study confirmed LncRNA MAGI2-AS3 could attenuate NSCLC progression by targeting the miR-629-5p/TXNIP axis [122]. In addition, the LHFPL3-AS2, a novel lncRNA, is significantly reduced in NSCLC tissues, resulting in more SFPQ binding to the TXNIP promoter and leading to TXNIP transcriptional inhibition, thereby ultimately promoting the migration and invasion of NSCLC cells [123]. Sodium butyrate (NaBu) shows promise in cancer therapy. TXNIP induced by NaBu regulates H4K5 acetylation and H3K4 trimethylation by increasing WDR5 expression, caspase 3/7 activation, and cell death [124]. In addition, the study has also demonstrated that NaBu-induced TXNIP also interacts with TNF receptor-related factor 6 (TRAF6) through its PPxY motif, thereby causing the change of TXNIP expression and its ubiquitination and affecting the migration and proliferation of the tumor cells in NSCLC [125].

Ubiquitin-like PHD and ring finger domain 1 (UHRF1) is an indispensable epigenetic regulator in the UHRF family. In renal cell carcinoma (RCC), UHRF1 recruits histone deacetylase 1 (HDAC1) into the TXNIP promoter, reducing TXNIP expression and promoting the progression of RCC [126]. circRNA also acts as an essential regulator in cancer, and circRNA rapef5 (crapgef5) targets the expression of Mir-27a-3p, which targets the 3′UTR in TXNIP to downregulate the expression of TXNIP, promoting the proliferation and migration of tumor cells in RCC [127]. TXNIP can also negatively regulate the progression of bladder carcinogenesis (BC) by inhibiting the extracellular signal-regulated kinase (ERK) induced by stromal cell-derived factor 1 or CXC chemokine receptor type 4 [128]. RNF2 has highly expressed in prostate cancer (PCA) tumor tissues. The researchers discovered that RNF2 regulates TXNIP expression through histone H2A ubiquitination, leading to cell cycle arrest, increased apoptosis, and inhibited cell proliferation [129]. Another study has reported that the protooncogene C-Myc can activate glutaminase 1 (GLS1) and reduce the activity of transcription factor MondoA, downregulating the expression of TXNIP and accelerating the proliferation of PCA cells [130].

### 4.4. Brain and Nervous System Diseases

**Figure 5:**
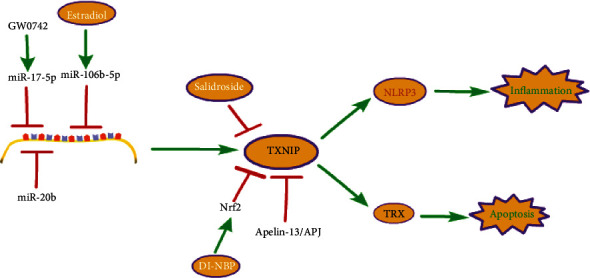
The main mechanism of TXNIP in the brain and nervous system diseases. TXNIP: thioredoxin-interacting protein; TRX: thioredoxin; NLRP3: NOD-like receptor family protein 3; Nrf2: nuclear factor erythrocyte 2-related factor 2; DI-NBP: Dl-3-n-butylphthalide.

More and more evidence shows that oxidative stress [131–133], mitochondrial dysfunction [134], changes in calcium homeostasis [135], and inflammatory response [136] are related to pathological changes in brain tissue. Studies have shown that TXNIP increases in neurodegenerative diseases and cerebrovascular diseases, including Alzheimer's disease (AD) [137], stroke [138], and subarachnoid hemorrhage (SAH) [139], which induces apoptosis and inflammatory of brain cells. In AD, the main pathological features are neurofibrillary tangles formed by abnormal accumulation of phosphorylated Tau in the cytoplasm and senile or neuritic plaques with *β* -amyloid protein as the central core surrounded by neurite or abnormal neuronal processes [140]. They continue to accumulate in the brain, triggering ER stress and leading to neurodegeneration [141]. The involvement of TXNIP in AD is mainly related to inflammation [142]. ER stress can promote the activation of TXNIP/NLRP3 inflammatory bodies in the hippocampus of the AD brain [141]. Early brain injury (EBI) is thought to be a main factor in the poor prognosis after SAH. Apoptosis is the primary pathological mechanism of EBI. TXNIP induced by protein kinase RNA like ER kinase (PERK) participates in EBI by promoting apoptosis [143]. Moreover, TXNIP can also participate in EBI after SAH by mediating inflammation [143]. Bakuchiol (Bak) has been proven to have multiple organ-protective effects. Bak reduces blood-brain barrier (BBB) damage, oxidative stress, and apoptosis by regulating TRX1/TXNIP expression and AMPK phosphorylation to weaken EBI after SAH [144]. Micro-RNAs like miR-17-5P [145], miR-106b-5p [146], and miR-20b [147], inhibits TXNIP expression, protecting brain cells. Thus, upregulation of mir-RNA appears to be a therapeutic target such as GW0742 [145] and estradiol [146], to protect brain cells from apoptosis and inflammation damage. Dl-3-n-butylphthalide (Dl-NBP) treatment inhibits TXNIP-NLRP3 interaction and NLRP3 inflammasome activation by upregulating nuclear factor erythrocyte 2-related factor 2 (Nrf2) [148]. Salidroside treatment can enhance cognitive ability by regulating the expression of TRX, TXNIP, and NF-*κ*B proteins [149]. In addition, the apelin-13/APJ system can show its neuroprotective effect after SAH in many ways. The authors showed that the combination of exogenous apelin-13 and APJ can reduce ER stress-mediated oxidative stress and neuroinflammation by inhibiting the AMPK/TXNIP/NLRP3 signal pathway [150]. The main mechanism of TXNIP in the brain and nervous system diseases is shown in Figure 5.

## 5. Conclusions and Future Development

As we all know, TXNIP plays an essential role in the pathophysiology of various diseases. Researchers found TXNIP is related to cancers [93, 95, 107, 108, 112–117, 120, 121, 124–131, 151–153], atherosclerosis [69], diabetes and its complications [47, 53, 54, 57], neurodegenerative diseases, and cerebrovascular diseases including Alzheimer's disease [137, 138], stroke [139], and subarachnoid hemorrhage [140]. But the role TXNIP played in different diseases is not necessarily the same. TXNIP acts as a culprit in diseases like diabetes, neurodegenerative diseases, and cerebrovascular diseases. The overexpressed TXNIP can lead to the progression of the diseases. However, TXNIP is indispensable. It contributes to inhibiting the proliferation and migration of cancer cells. Low TXNIP concentration inhibits apoptosis, leading to cancer growth and migration. Thus, the TXNIP is a double-edged sword for us, and the balance of the TXNIP is crucial.

Furthermore, the pathophysiological role of TXNIP is closely associated with the response to ROS. ROS are byproducts of the normal metabolism of oxygen and present at low and stationary levels in normal cells [154]. Howbeit, ROS is nearly omnipresent and is related to plenty of diseases. Such as aging, the oxidative damage initiated by ROS is a major contributor to the functional decline according to the free radical theory of aging [155]. Besides, ROS is largely generated during inflammation, and the generated ROS will activate TXNIP even in normal cells. Based on that, we speculate that TXNIP might be involved in every disease related to ROS production other than the diseases mentioned above.

TXNIP can be used as a promising target for treatment. For cancers, as summarized previously, the concentration of TXNIP is low in cancers compared with normal cells, and downregulation of TXNIP indicates a poor prognosis [95]. What is more, TXNIP binds to PARP within the nucleus, and ROS-activated PARP will bind even tighter with TXNIP, preventing it from being released. Thus, low-leveled TXNIP might be a resistant mechanism for tumors to survive the radiotherapy. Hence, TXNIP may be a good target to treat cancers by increasing its level within cancer cells. Researchers found that vitamin D_3_ and sodium butyrate can increase TXNIP expression and decrease ubiquitin degradation, respectively, to increase TXNIP levels [125]. Those two drugs might be used as adjuvant therapies to treat cancers. Moreover, novel techniques may provide us with some inspiration, such as lipid nanoparticles (LNPs). LNPs are promising in vivo delivery vehicles that are widely used in many clinical trials. They can be designed to deliver mRNAs into cancer cells, consequently increasing the TXNIP level in cancer cells and causing cancer cell death. As for diseases like diabetes, atherosclerosis, and neurodegenerative diseases, which were caused by overexpressed TXNIP and consequently overactive apoptotic activity, TXNIP may still be an encouraging target. miRNAs can downregulate gene expressions by tightly binding to their corresponding mRNAs and subsequently degrading mRNAs. Ergo, we may use LNPs to deliver miRNAs into targeted cells to reduce the TXNIP expression and lower the TXNIP levels to rescue the cells. However, all of the strategies we mentioned above stay at the research level, and whether they can be used for clinical practice is unknown. Animal experiments and clinical trials should be done to determine their efficacy and safety.

Except for its role as a treatment target, TXNIP may be a good candidate as a biomarker. In people with diabetes, a high serum glucose level can cause endothelial dysfunction. The TXNIP pathway partially mediates endothelial dysfunction, and the TXNIP level is associated with the progression of endothelial dysfunction. As a result, TXNIP may be a marker for the severity of the injury. TXNIP also has the capability of being a biomarker to predict prognosis in various cancers. Downregulation of TXNIP indicates a poor prognosis in breast cancers. Furthermore, as TXNIP exists in nearly every cell and plays almost the same function in each cell, its expression may also indicate the prognosis of other cancers. Clinical studies may be done to reveal the availability of TXNIP as a good marker for the severity of diseases.
